# Pivotal Role of Toll-Like Receptors 2 and 4, Its Adaptor Molecule MyD88, and Inflammasome Complex in Experimental Tubule-Interstitial Nephritis

**DOI:** 10.1371/journal.pone.0029004

**Published:** 2011-12-14

**Authors:** Matheus Correa-Costa, Tarcio Teodoro Braga, Patricia Semedo, Caroline Yuri Hayashida, Luiz Roberto Grassmann Bechara, Rosa Maria Elias, Claudiene Rodrigues Barreto, Claudia Silva-Cunha, Meire Ioshie Hyane, Giselle Martins Gonçalves, Patricia Chakur Brum, Clarice Fujihara, Roberto Zatz, Alvaro Pacheco-Silva, Dario S. Zamboni, Niels Olsen Saraiva Camara

**Affiliations:** 1 Laboratory of Transplantation Immunobiology, Department of Immunology, Institute of Biomedical Sciences IV, University of São Paulo, São Paulo, Brazil; 2 Laboratory of Clinical and Experimental Immunology, Nephrology Division, Federal University of São Paulo, São Paulo, Brazil; 3 School of Physical Education and Sport, University of Sao Paulo, São Paulo, Brazil; 4 Renal Division, Department of Clinical Medicine, Faculty of Medicine, University of São Paulo, São Paulo, Brazil; 5 Instituto Israelita de Ensino e Pesquisa Albert Einstein. Renal Transplantation Unit, Albert Einstein Hospital, São Paulo, Brazil; 6 Department of Cell Biology, Medical School Ribeirão Preto, Universidade de São Paulo, São Paulo, Brazil; Duke University Medical Center, United States of America

## Abstract

Tubule-interstitial nephritis (TIN) results in decreased renal function and interstitial inflammation, which ultimately leads to fibrosis. Excessive adenine intake can cause TIN because xanthine dehydrogenase (XDH) can convert this purine into an insoluble compound, which precipitates in the tubuli. Innate immune sensors, such as Toll-like receptors (TLR) and inflammasome complex, play a crucial role in the initiation of inflammation. The aim of this study was to evaluate the roles of TLR-2 and -4, Myd88 and inflammasome complex in an experimental model of TIN. Here, we show that wild-type (WT) mice fed adenine-enriched food exhibited significant renal dysfunction and enhanced cellular infiltration accompanied by collagen deposition. They also presented higher gene and protein expression of pro-inflammatory cytokines. In contrast, TLR-2, -4, MyD88, ASC and Caspase-1 KO mice showed renoprotection associated with expression of inflammatory molecules at levels comparable to controls. Furthermore, treatment of WT animals with allopurinol, an XDH inhibitor, led to reduced levels of uric acid, oxidative stress, collagen deposition and a downregulation of the NF-kB signaling pathway. We concluded that MyD88 signaling and inflammasome participate in the development of TIN. Furthermore, inhibition of XDH seems to be a promising way to therapeutically target the developing inflammatory process.

## Introduction

Tubule-interstitial nephritis (TIN) is a common, but underestimated, disease characterized by acute inflammatory infiltrates associated with deterioration in renal function. If the causative stimulus persists, the disease process can worsen and cause fibrosis deposition and tubular damage [1]. Adenine-enriched food is an experimental model of TIN in which there is an excess of this purine, thus allowing it to become a substrate for xanthine dehydrogenase (XDH). XDH converts adenine to 2,8-dihydroxyadenine (DHA), an insoluble compound that precipitates in the tubule-interstitial compartment, and causes nephrolithiasis followed by extensive tubular dilation, necrosis and apoptosis [2], [3]. Consequently, the presence of damaged tissue initiates an intense inflammatory process, which apparently contributes to the progression of the disease [4].

Toll-like receptors (TLRs) are sensors of the innate immune system that recognize pathogen-associated molecular patterns and injured tissue signals, which are called damage-associated molecular patterns (DAMPs). Activation of TLRs induces a pro-inflammatory cascade, with downstream participation of NF-κB target genes [5]. Furthermore, the activation of intracellular sensors such as NOD-like receptors, for example, NLRP3, leads to the formation of the inflammasome complex by converting pro-caspase-1 into active caspase-1, which in turn results in secretion of IL-1β, IL-18 and IL-33 [6]. The adaptor molecule ASC plays an important role in this process because it recruits activated NLRP3 and caspase-1 to form the inflammasome complex [7]. These innate immune elements have been widely recognized to be some of the molecules involved in acute and chronic kidney diseases[4], [8], [9], however, it is still unclear whether they actively participate in the development of TIN.

Therefore, we hypothesize that TLR-2, -4 and MyD88, as well as inflammasome complex elements, play an important role in our experimental model of TIN.

## Results and Discussion

**Figure 1:**
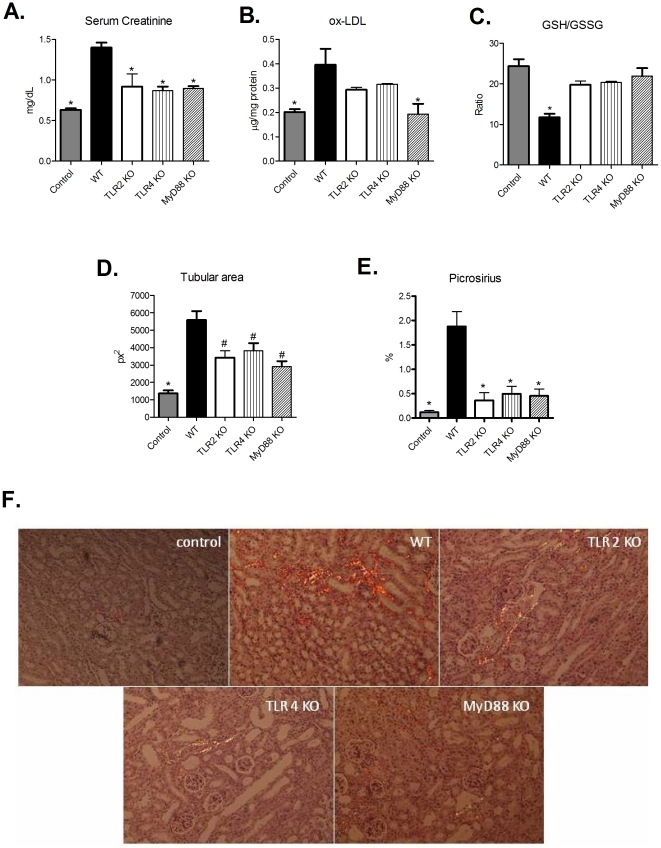
Lack of TLR signaling protects mice from TIN. (A) Renal function was assessed by serum creatinine levels from control, WT and TLR2, -4 and Myd88 KO mice (ANOVA test - p = 0.0001, with Tukey post test * p = 0.0001 vs WT group) (B) Detection of ox-LDL in renal tissue was used as an indirect measure of oxidative stress (ANOVA test - p = 0.0119, with Tukey post test * p<0,05 vs WT group). (C) GSH/GSSG ratio was also used as an index of oxidative stress. (ANOVA test – p<0.0001, with Tukey post test * p<0.001 vs. control, TLR2 KO, TLR4 KO and MyD88 KO groups). (D) Tubular dilation was quantified in WT and KO animals, and is expressed as tubular area (ANOVA test - p = 0.0014, with Tukey post test * p<0.001 vs WT group and # p<0.01 vs WT group). (E) Graphic quantification of fibrosis deposition observed by picrosirius staining (ANOVA test - p = 0.0001, with Tukey post test *p<0.001 vs. WT group). Images obtained by polarized light microscopy are shown in panel (F). n = 5 animals/group.

Initially, we observed that wild type (WT) animals that received adenine-supplemented food exhibited an enhancement in XDH, TLR-2, -4, MyD88, and NLRP3 and gene expression (Figure S1) and, as observed by others [3], [10], these animals had increased serum creatinine levels, cellular infiltration, tubular dilation and fibrosis deposition (Figure 1). Next, we provided adenine-supplemented food to TLR-2, -4 and MyD88 KO mice. The food intake was not different between WT or KO mice (data not shown). As observed in Figure 1, all the KO animals exhibited a striking protection of renal function and less oxidative stress, as detected by reduced levels of ox-LDL and GSSG/GSH ratio, compared with WT mice on the same diet. Also, KO mice exhibited significantly less inflammatory cellular infiltrations, tubular dilation and collagen deposition (Figure 1 and S1).

**Figure 2:**
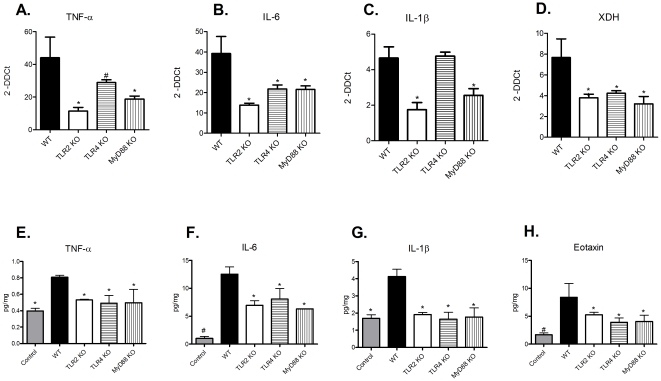
The inflammatory process resulting from TIN is decreased in TLR 2, -4, and MyD88 KO mice. Kidney samples from WT and TLR2, -4 and MyD88 KO animals were processed to determine the levels of (A) TNF-α (ANOVA test - p = 0.0190, with Tukey post test *p<0.01 vs WT group and #p<0.05 vs. WT group), (B) IL-6 (ANOVA test - p = 0.0416, with Tukey post test *p<0.05 vs. WT group), (C) IL-1β (ANOVA test - p = 0.0010, with Tukey post test * p<0.01 vs. WT group), and (D) XDH genes (ANOVA test - p = 0.048, with Tukey post test - *p<0.05 vs. WT group). Gene expression from control animals was assigned a value of 1. Protein levels of (E) TNF-α (ANOVA test - p = 0.0021, with Tukey post test, with Tukey post test *p<0.05 vs. WT group), (F) IL-6 ANOVA test - p = 0.0001, with Tukey post test *p<0.05 vs. WT group and #p<0.001 vs WT group), (G) IL-1β (ANOVA test - p = 0.0059, with Tukey post test * p<0.01 vs. WT group) and (H) eotaxin (ANOVA test - p = 0.0184, with Tukey post test *p<0.05 vs. WT group and # p<0.01 vs. WT group) were also assessed in these animals. n = 5 animals/group.

Next, we investigated what molecular mechanism could be involved in this process. We observed that the TLR-2 and MyD88 KO animals exhibited significantly less TNF-α, IL-6 and IL-1β at both gene and protein levels compared with WT animals. Interestingly, we didńt observe a reduction in gene expression of IL-1β in TLR-4 KO group, but the protein expression was decreased. As we looked both expressions at the same time point, we can suggest that the gene expression had formerly been reduced in these mice, which lately led to less protein expression of such molecule, as observed in our analysis (Figure 2, panels A-C and E-G). KO animals also showed decreased amounts of eotaxin, a chemmoattractant molecule for eosinophils (Figure 2H). The eosinophil population has been highly implicated in TIN [1]. Although we have observed very little eosinophils in the renal tissue of WT animals, KO animals displayed no eosinophils entrance. Probably, the increase in eotaxin will culminate in eosinophil infiltration in a posterior time point. Our data provides evidence that there is a chemotaxis signaling for this immune cellular type. Finally, the KO animals displayed significantly lower expression of XDH (Figure 2D). These data corroborate another study that showed enhanced XDH gene expression in kidney epithelial cells following exposure to various cytokines, including TNF-α, IFN-γ, IL-1β and IL-6 [11]. Together, these results indicate that MyD88 signaling seems to be involved in the inflammatory response observed in TIN.

**Figure 3:**
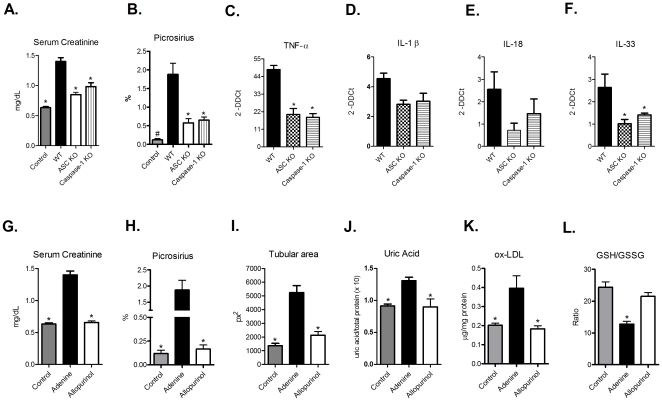
Inflammasome elements are important for TIN progression, and inhibition of the xanthine oxidoreductase system attenuates the development of this disease. Control, WT and ASC and Caspase-1 KO animals were evaluated for (A) serum creatinine levels (ANOVA test - p<0.0001, with Tukey post test – p<0.001 vs. WT group), (B) fibrosis deposition by picrosirius staining (ANOVA test - p<0.0001, with Tukey post test #p<0.0001 vs. WT group and *p<0.01 vs WT group), and gene expression of (C) TNF-α (ANOVA test - p = 0.0002, with Tukey pos test *p<0.001 vs. WT group), (D) IL-1β (ANOVA test - p = 0,0785), (E) IL-18 (ANOVA test - p = 0.1792) and (F) IL-33 (ANOVA test - p = 0.0364, with Tukey post test *p<0.05 vs. WT group). In addition, allopurinol-treated animals exhibited renoprotection, as demonstrated by decreased serum creatinine levels (G) (ANOVA test - p<0.0001, with Tukey post test *p<0.01 vs. adenine group), fibrosis deposition (H) (ANOVA test - p<0.0001, with Tukey post test *p<0.001 vs. adenine group), tubular dilation (I) (ANOVA test - p = 0.0001, with Tukey post test *p<0.01 vs. adenine group), tissue uric acid levels (J) (ANOVA test p = 0.0159, with Tukey post test *p<0.05 vs. adenine group), presence of ox-LDL (K) (ANOVA test - p = 0.0211, with Tukey post test, *p<0.05 vs. adenine group) and (L) GSH/GSSG ratio (ANOVA test - p = 0.0002, with Tukey post test *p<0.001 vs. control and allopurinol groups). n = 5 animals/group.

Because we observed enhanced gene expression of NLRP3 in WT animals fed with an adenine-enriched diet, we hypothesized that inflammasome complex activation also participates in the development of TIN. To examine this hypothesis, we fed ASC and caspase-1 KO animals with adenine-supplemented food. As expected, we observed that renal function was significantly better in the KO mice compared to that in their WT counterparts (Figure 3A). In addition, mRNA levels of TNF-α, IL-1β, IL-18, and IL-33 were decreased in the KO animals compared with WT mice (Figure 3, panels C–F). Histological evaluation revealed that there were less inflammatory infiltrates and collagen deposits in the mice lacking ASC and caspase-1 compared to WT mice (Figure S2, panels A and B and Figure 3B). Together, these results indicate that activation of inflammasome complexes can exacerbate the renal injury caused by TIN by promoting the secretion of inflammatory cytokines.

In addition to producing DHA, the metabolism of purines produces hypoxanthine, which is subsequently converted into xanthine and uric acid (and accompanied by the release of reactive oxygen species) by xanthine oxidoreductase (XOR), an enzymatic complex consisting of XDH and xanthine oxidase (XO) [12]. XDH can be converted into XO in the presence of calcium and/or calpain [13]. These purine breakdown products can have strong effects on innate immune signaling because reactive oxygen species can serve as a second messenger and lead to NF-κB activation [14], and uric acid can be sensed by NLRP3, which results in activation of the inflammasome complex [15]. Allopurinol has been shown to inhibit XDH and XO, which results in decreased renal injury and subsequent kidney protection [16]. To determine whether inhibition of these enzymes by allopurinol could modulate the inflammatory process in TIN, we fed WT animals with adenine-supplemented food and treated them with allopurinol in the drinking water. The treatment effectively inhibited the XOR system, as observed by decreased gene expression of XDH (Figure S2, panel C). Also, the allopurinol-treated group showed impressive renoprotection and lower TLR-2, -4, and MyD88 gene expression, compared with animals that only fed the adenine-supplemented food (Figures S2, panels D-F). Serum creatinine levels, inflammatory infiltrates, tubular dilation and fibrosis deposition were all significantly decreased in allopurinol group, with values markedly similar to control animals (that received standard food) (Figure S2, panel A and Figure 3, panels G-I). Moreover, we observed significant increase of uric acid levels, ox-LDL and GSSG/GSH ratio in the adenine group, but these measurements were completely restored in the allopurinol-treated group, with similar values of the control group (Figure 3, panels J, K and L).

**Figure 4:**
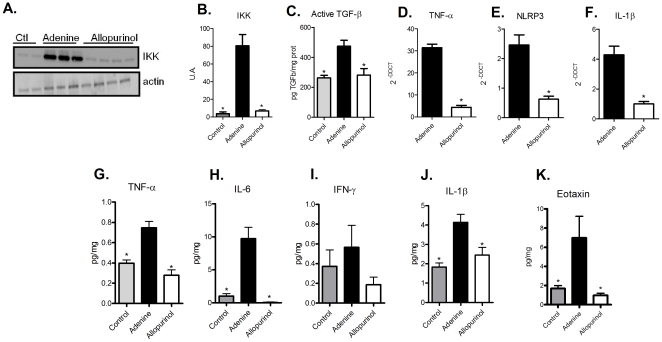
Treatment with allopurinol reduces inflammatory and fibrotic signaling in mice fed an adenine-rich diet. Renal tissue samples from control, adenine and allopurinol treated animals were processed to examine gene and protein expression of pro-inflammatory and pro-fibrotic molecules. (A) Representative image of an IKK western blot. Actin was used as a loading control. (B) Graphic quantification of bands expressed from panel (A) (ANOVA test - p<0.0001, with Tukey post test *p<0.0001 vs. adenine group). (C) TGF-β1 active molecule levels were dosed on control, adenine and allopurinol groups (ANOVA test - p = 0.0047, with Tukey post test *p<0.001 vs. adenine group). The expression of inflammatory genes was determined by measuring the levels of TNF-α (student t test * p<0.0001 vs. adenine group), NLRP3 (student t test *p = 0.0007 vs. adenine group) and IL-1β (student t test * p<0.0005 vs. adenine group) – panels D, E and F, respectively. Also, protein expression of TNF-α (G) (ANOVA test - p = 0.0031, with Tukey post test *p<0.01 vs. adenine group), IL-6 (H) (ANOVA test - p = 0.0015, with Tukey post test *p<0.001 vs. adenine group), IFN-γ (I) (ANOVA test - p = 0.3849), IL-1β (J) (ANOVA test - p = 0.0095, with Tukey pos test *p<0.05 vs. adenine group) and eotaxin (K) (ANOVA test - p = 0.0117, with Tukey pos test *p<0.05 vs. adenine group) were assessed. n = 5 animals/group.

The renal protection induced by allopurinol involves the NF-κB pathway, as animals that received both adenine-supplemented food and allopurinol showed reduced IKK expression, with levels comparable to control animals (Figure 4, panels A and B).

Analysis of collagen deposition revealed that excessive adenine intake induced a chronic phase of TIN, with fibrosis deposition (Figure S2, panel A and Figure 3H). TGF-β plays a crucial role in fibrosis [17], so we wondered whether this cytokine was involved in experimental TIN. As shown in Figure 3C, we observed that in the adenine-supplemented group, there was a significantly higher expression of TGF-β compared with controls, and that this increase was abrogated by allopurinol treatment. In reality, we also observed significantly lower levels of TNF-α, NLRP3, IL-1β, and IL-18 and genes in the allopurinol-treated group (Figure S2, panel G and Figure 4, panels D-F), as well as lower levels of pro-inflammatory proteins (Figure 4, panels G-K). A recent paper demonstrated that in an experimental model of lung injury, the administration of allopurinol can inhibit inflammation and fibrotic processes [18]. Together, these results indicate that adenine metabolism end-products can result in the production of pro-inflammatory molecules that can activate some innate immune pathways. Furthermore, the administration of allopurinol reduces this process, thereby decreasing the innate immune signaling.

As observed previously in different models, the presence of an inflammatory environment can be a key factor influencing the development of renal fibrosis [19], [20]. Our data confirm this idea in an adenine injury model and provide experimental support for a link between acute and chronic TIN. A recent review states that the inflammatory response that occurs after an acute kidney injury leads to progressive renal disease. Modulating the inflammatory process could be extremely beneficial in inhibiting fibrosis deposition [21].

It is important to highlight that our experimental model represents a specific situation of TIN (excessive purine) and such results may not be completely applicable for all situations of nephritis. Other models should be used to evaluate the role of innate immune receptors in different situations of TIN.

Our results indicate, for the first time, that some intra- and extracellular innate immune receptors clearly participate in the model of excessive purine TIN. We also propose that allopurinol, a widely used drug, can reduce some of the factors released after activation of these receptors. This fact provides a new perspective on the clinical use of this drug. Further studies are necessary to completely elucidate the cellular mechanisms underlying these processes and to help find new therapeutic targets that inhibit the progression of this renal disease.

## Methods

### Animals and reagents

TLR-2, -4 and MyD88 genetically deficient mice (KO) mice were originally obtained from S Akira (Osaka University). ASC KO and caspase-1 KO mice were purchased from School of Medicine of Ribeirão Preto, University of Sao Paulo. C57BL/6 (WT) mice were obtained from our Isogenic Breeding Unit (Immunology Department, Biomedical Science Institute, University of Sao Paulo). The knockout mice have the C57BL/6 genetic background and were backcrossed for 20 generations. All animals were used at 8–12 wk of age. Animals were fed with 0.25% adenine enriched food (Rhoster, Aracoiaba da Serra, Brazil) for 10 days and were sacrificed thereafter. Control animals received standard food for the same period. For allopurinol experiments, WT animals received adenine enriched food and allopurinol (Sigma, St Louis, USA) were diluted in drinking water (150 mg/mL) and given ad libitum. All experimental procedures were done in accordance to ethical statements approved by the institutional Ethical Committee of the University of Sao Paulo. The study was approved by the Ethical Comittee after detailed analysis (document number 118/2008).

### Biochemical parameter analyses

Blood was collected for serum creatinine measurements, and kidneys harvested for uric acid dosage. All samples were analyzed by colorimetric assays using commercially-purchased kits (Creatinine and Uric Acid Kits, Labtest, Minas Gerais, Brazil).

### Determination of GSH/GSSG ratio

A glutathione fluorescent detection kit (Arbor Assays LLC, Ann Arbor, Michigan) was used to measure reduced/oxidized glutathione (GSH/GSSG) ratio in kidney samples. Kidney cells were lysed in ice cold 100mM phosphate buffer, pH 7, and centrifuged at 14,000 rpm for 10 minutes at 4°C. Proteins were precipitated with an equal volume of ice cold 5% (w/v) 5-sulfosalicylic acid (SSA) solution. After mixing the sample or standard with a proprietary nonfluorescent molecule, ThioStar®, that covalently bind to the free thiol group on GSH to yield a highly fluorescent product, and incubating at room temperature for 15 minutes, the fluorescent product was read at 510 nm in a fluorescent plate reader with excitation at 390 nm. Free glutathione, GSH, was read first, followed by addition of a reaction mixture that converts all the oxidized glutathione, GSSG, into free GSH, which then reacts with the excess ThioStar® to yield the signal related to total GSH content. The concentration of GSSG was estimated by subtracting the measured free GSH from the measured total GSH. GSH/GSSG ratio was calculated as an index of oxidative stress (lower index indicates increased oxidative stress).

### Histomorpmometric analyses

Formaldehyde-fixed paraffin sections of the kidneys were stained with Hematoxilin-Eosin and Picrosirius for evaluation of cellular infiltration, tubular dilation and fibrosis deposition. Renal histomorphometric analyses were made by two “blinded” renal histologists. Tubular area was assessed by quantification of tubular spaces, and results are expressed as pixels^2^. Picrosirius stained sections were analyzed by an Olympus BX50 microscope with an Olympus camera attached (USA). Manual shots were taken of the cortex, magnified 40X, and observed under polarized light. Photos of at least 5 different fields in each slide were taken, and structures such as the glomeruli, subcapsular cortex, large vessels and medulla were excluded. The pictures were digitalized (HP Scanjet 2400) and then the interstitial volume of collagen in the cortex compared to the overall cortex area was quantified by morphometry. For the morphometric analysis, the Image Processing and Analysis in Java, Image J software was used. The result of the analysis is represented by percentage, and refers to the proportion of the interstitial volume of collagen in the cortex to the total cortical interstitial volume, and then the arithmetic mean of the analyzed fields was calculated for each slide.

### Gene Profiles

Kidney samples were snap-frozen in liquid nitrogen. Total RNA was isolated from kidney tissue using the TRIzol Reagent (Invitrogen, Carlsbad, USA) and protocol according to Invitrogen. RNA concentrations were determined by spectrophotometer readings at absorbance 260 nm. First-strand cDNAs were synthesized using the MML-V reverse transcriptase (Promega, Madison, USA). RT-PCR was performed using the Taqman real-time PCR assay (Applied Biosystem, USA) for the following molecules: HPRT (Mm00446968_m1), TNF-α (Mm00443258_m1), IL-1β (Mm00434228_m1_m1), IL-6 (Mm004461690_m1), TLR2 (Mm00442346_m1), TLR4 (Mm00445273_m1), MyD88 (Mm00440338_m1), XDH (Mm00442110_m1), IL-18 (Mm00434226_m1), IL-33 (Mm00505403_m1) and NLRP3 (Mm00840904_m1). Cycling conditions were as follows: 10 minutes at 95°C followed by 45 cycles at 20 seconds each at 95°C, 20 seconds at 58°C, and 20 seconds at 72°C. Analysis used Sequence Detection Software 1.9 (SDS). mRNA expression was normalized to HPRT expression.

### Elisa Assay for TGF-β

Total TGF-β1 protein was measured by ELISA (TGFβ1 Emax®, Promega, Madison, USA). Kidney cells were lysed in RIPA buffer and protein levels quantified by DC Protein Assay (Bio-Rad, Hercules, USA). After overnight coating of a 96-well plate with a primary antibody, TGF-β1 was detected in cell lysates using a secondary antibody. The system uses horseradish peroxidase-conjugated secondary antibody and a single-component TMB substrate for the final chromogenic detection of bound TGF-β1. Using this assay, biologically active TGFβ-1 can be detected in the range of 15.6–1,000 pg/ml. Results are expressed as ng/mg of TGF-β protein.

### Western blot analysis

Kidney cells were lysed in RIPA buffer, run on a 10% SDS-polyacrylamide electrophoresis gel and transferred onto a nitrocellulose membrane (Hybond C Extra, Amershan Biosciences, Little Chalfon, USA). Membranes were incubated with primary rabbit anti–mouse IKK (Santa Cruz Biotechnology, Santa Cruz, USA) antibody, using manufacturer-recommended dilutions, followed by a peroxidase-conjugated mouse anti-rabbit IgG antibody (Sigma, St. Louis, USA). HRP activity was detected using enhanced chemiluminescence. The membrane was stripped and probed with mouse primary anti-β-actin antibody (Sigma, St. Louis, USA) to confirm and estimate the loading and the transfer. We used the software, GeneSnap (Syngene, USA) and Gene Tools (Syngene, USA), to analyze the bands.

### Bioplex

Kidney cells were lysed in RIPA buffer with protease inhibitor. A Bio-Plex mouse Plex cytokine assay kit (Bio-Rad Laboratories, Inc., Hercules, CA, USA) was used to test samples for the presence of 15 cytokines. The assay was read on the Bio-Plex suspension array system, and the data were analyzed using Bio-Plex Manager software version 4.0. Standard curves ranged from 32,000 to 1.95 pg/mL.

### Determination of anti-oxLDL

A 96-well ELISA plate was coated with 50 µl of the oxLDL [7.5 µg/ml per well] in 0.1 mol/l carbonate/bicarbonate buffer (pH 9.6) and left overnight at 4°C. After washing with PBS, the plate was blocked with 3% gelatin at room temperature for 24 h. Tissue homogenate samples (50 µl) were diluted 1∶400 before addition to the wells. After 2 h incubation, the plate was washed with PBS containing 0.05% Tween, and peroxidase-conjugated goat anti-human IgG (dilution 1∶1000 — Kirkegaard & Perry Laboratories, Gaithersburg, MD) was added. After washing, tetra-methyl-benzidine (250 µl 3,3′5,5′ 6.5% in DMSO), plus H2O2 in Citrate phosphate buffer, (0.1 mol/l, pH 5.5) were added as substrate. The reaction was stopped by the addition of 2 mol/l H2SO4 and measured at 450 nm in optical density (OD). Results are expressed as ng/mg of ox-LDL protein.

### Statistics

The data were described as mean ± S.E.M. Differences among groups were compared using ANOVA (with Tukey post-test) and Student t-test. Significant differences were regarded as p<0.05. All statistical analyses were performed with the aid of GraphPad PRISM®.

## Supporting Information

Figure S1 **Adenine model of TIN increases expression of innate immune receptors in wild type animals.** C57/Bl6 mice were fed either with standard or adenine-enriched food, and were sacrificed after 10 days. Kidney samples were collected to determine expression levels of XDH (A) (student t test *p = 0.0258 vs. control group), TLR2 (B) (student t test *p = 0.0047 vs. control group), TLR4 (C) (student t test p = 0.2763 vs. control group), Myd88 (D) (student t test *p = 0.0213 vs. control group), and NLRP3 (E) (student t test *p = 0.0244 vs. control group) genes. Representative pictures of kidney tissue stained with HE and picrosirius from these animals are shown in panel (F). n = 5 animals/group.
(TIF)

Figure S2 **ASC KO, Caspase-1 KO and allopurinol-treated mice show renoprotection and decreased inflammation. (**A) Representative images of renal tissue stained with HE and picrosirius obtained from control, WT, ASC KO, Caspase-1 KO and allopurinol-treated mice. (B) Tubular dilation, quantified by tubular area, shown from control, WT, ASC KO and Caspase-1 KO animals (ANOVA test - p = 0.0005, with Tukey post test, #p<0.001 vs. WT group and * p<0.05 vs WT group). Kidney samples from WT and allopurinol-treated animals were processed to determine gene expression levels of (C) XDH (student t test *p = 0.0323 vs. adenine group), (D) TLR2 (student t test *p = 0.0047 vs. adenine group), (E) TLR4 (student t test *p = 0.0474 vs. adenine group), (F) MyD88 (student t test *p = 0.0372 vs. adenine group), (G) and IL-18 (student t test p = 0.3717 vs. adenine group). Expression levels from control animals were given a value of 1. n = 5 animals/group.
(TIF)
