# Supplementary figures and images for: Pivotal Role of Toll-Like Receptors 2 and 4, Its Adaptor Molecule MyD88, and Inflammasome Complex in Experimental Tubule-Interstitial Nephritis
Source: PLoS One. 2011 Dec 14;6(12):e29004. doi: 10.1371/journal.pone.0029004 (PMC3237574; doi:10.1371/journal.pone.0029004)

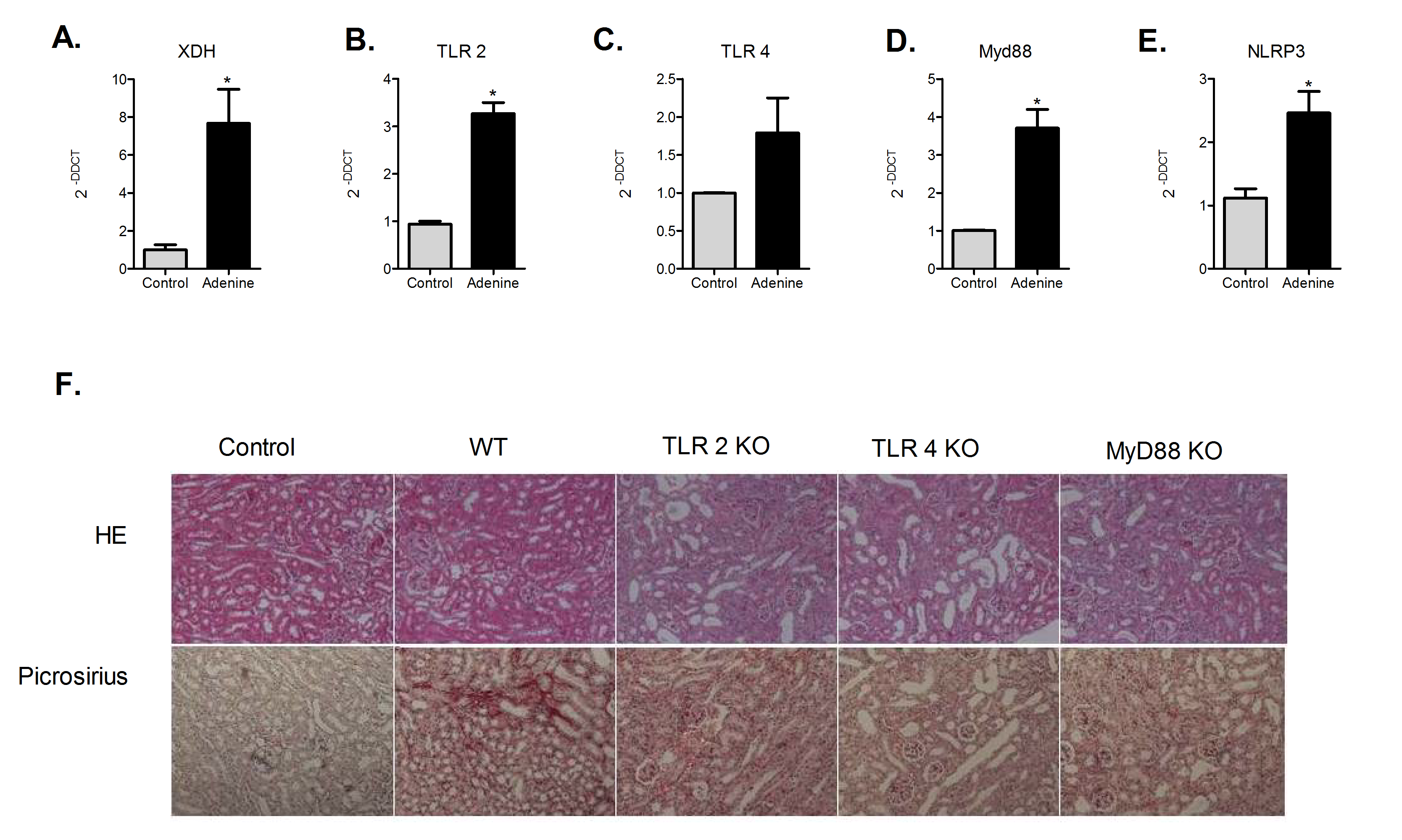

Supplement: Figure S1 — Adenine model of TIN increases expression of innate immune receptors in wild type animals. C57/Bl6 mice were fed either with standard or adenine-enriched food, and were sacrificed after 10 days. Kidney samples were collected to determine expression levels of XDH (A) (student t test *p = 0.0258 vs. control group), TLR2 (B) (student t test *p = 0.0047 vs. control group), TLR4 (C) (student t test p = 0.2763 vs. control group), Myd88 (D) (student t test *p = 0.0213 vs. control group), and NLRP3 (E) (student t test *p = 0.0244 vs. control group) genes. Representative pictures of kidney tissue stained with HE and picrosirius from these animals are shown in panel (F). n = 5 animals/group. (TIF) [file pone.0029004.s001.tif]

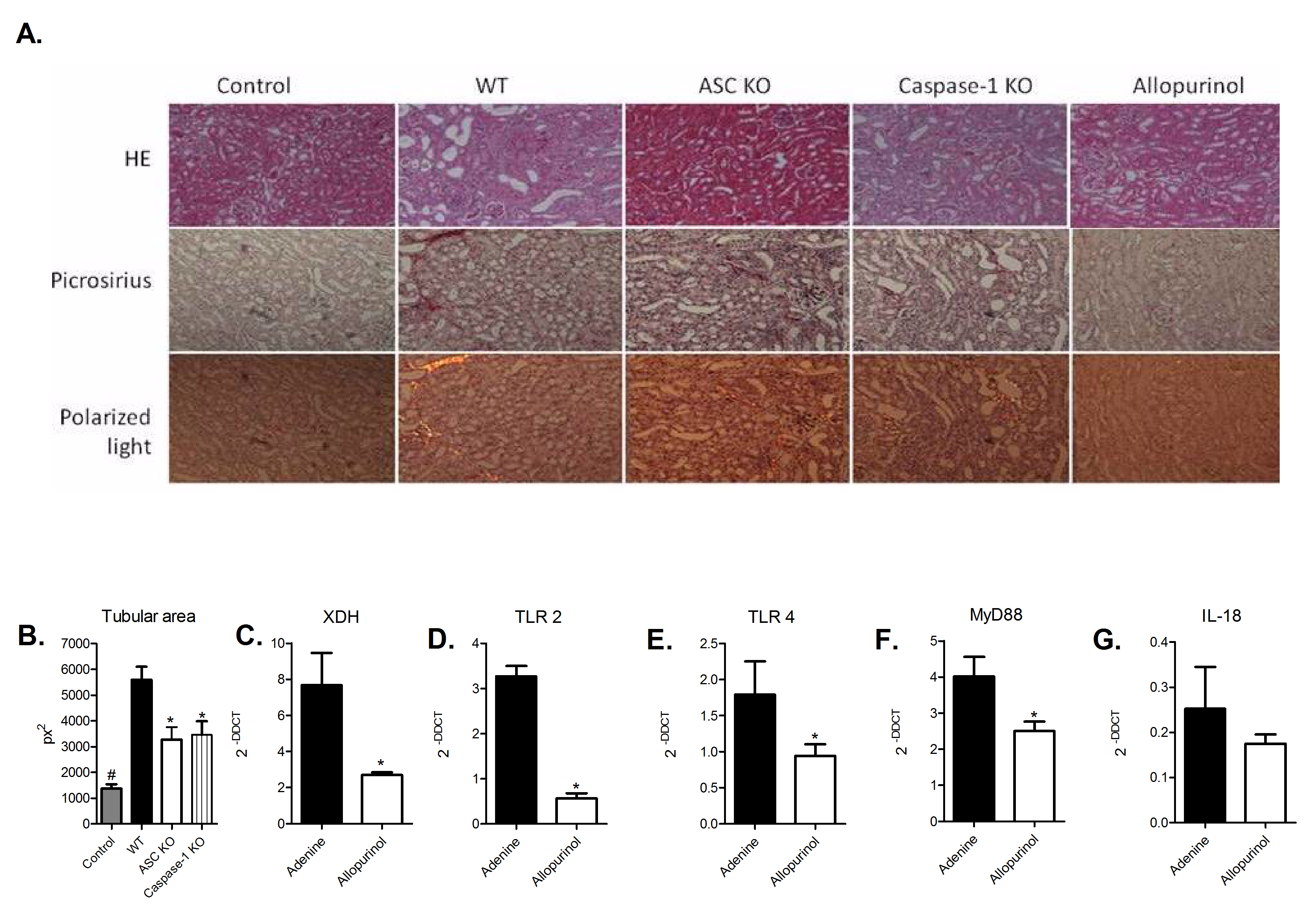

Supplement: Figure S2 — ASC KO, Caspase-1 KO and allopurinol-treated mice show renoprotection and decreased inflammation. (A) Representative images of renal tissue stained with HE and picrosirius obtained from control, WT, ASC KO, Caspase-1 KO and allopurinol-treated mice. (B) Tubular dilation, quantified by tubular area, shown from control, WT, ASC KO and Caspase-1 KO animals (ANOVA test - p = 0.0005, with Tukey post test, #p<0.001 vs. WT group and * p<0.05 vs WT group). Kidney samples from WT and allopurinol-treated animals were processed to determine gene expression levels of (C) XDH (student t test *p = 0.0323 vs. adenine group), (D) TLR2 (student t test *p = 0.0047 vs. adenine group), (E) TLR4 (student t test *p = 0.0474 vs. adenine group), (F) MyD88 (student t test *p = 0.0372 vs. adenine group), (G) and IL-18 (student t test p = 0.3717 vs. adenine group). Expression levels from control animals were given a value of 1. n = 5 animals/group. (TIF) [file pone.0029004.s002.tif]
